# Dose–response effects for depression and Schizophrenia management on hospital utilization in Illinois Medicaid: a multivariate regression analysis

**DOI:** 10.1186/1472-6963-14-288

**Published:** 2014-07-03

**Authors:** Gregory D Berg, Shawn Donnelly, Kathleen Warnick, Wendie Medina, Mary Miller

**Affiliations:** 1 McKesson Corporation, Westminster, CO, USA; 2 Illinois Department of Healthcare and Family Services, Springfield, IL, USA; 3 Formerly with Illinois Department of Healthcare and Family Services, Springfield, IL, USA

## Abstract

**Background:**

The prevalence of schizophrenia and depression in the United States is far higher among Medicaid recipients than in the general population. Individuals suffering from mental illness, including schizophrenia and depression, also have higher rates of emergency department utilization, which is costly and may not generate the positive health outcomes desired. Disease management programs strive to help individuals suffering from chronic illnesses better manage their condition(s) and seek health care in the appropriate settings. The objective of this manuscript is to estimate a dose–response impact on hospital inpatient and emergency room utilizations for any reason by Medicaid recipients with depression or schizophrenia who received disease management contacts.

**Methods:**

Multivariate regression analysis of panel data taken from administrative claims was conducted to test the hypothesis that increased contacts lower the likelihood of all-cause inpatient admissions and emergency room visits. Subjects included 6,274 members of Illinois’ non-institutionalized Medicaid-only aged, blind or disabled population diagnosed with depression or schizophrenia. The statistical measure is the odds ratio. The odds ratio association is between the monthly utilization indicators and the number of contacts (doses) a member had for each particular disease management intervention.

**Results:**

Higher numbers of intervention contacts for Medicaid recipients diagnosed with depression or schizophrenia were associated with statistically significant reductions in all-cause inpatient admissions and emergency room utilizations.

**Conclusions:**

There is a high correlation between depression and schizophrenia disease management contacts and lowered all-cause hospital inpatient and emergency room utilizations.

## Background

The presence of mental illness is associated with substantially higher per capita costs and all-cause hospitalization rates compared to the general population [1]. Mental disorders are among the five most costly health conditions to treat and manage. In 2005, national expenditures for the treatment of mental health and substance abuse disorders were tallied at $135 billion, [2] and costs are expected to grow to $239 billion by 2014 [3]. Of the 95 million visits to U.S. emergency rooms in 2007, nearly 12 million visits, or one in eight, involved people with a mental disorder, substance abuse problem, or both. Reflecting the fact that Medicaid is the single largest payer for mental health services in the U.S., [4] Medicaid was billed about 20 percent of the time for these visits. Nearly 41 percent of mental disorder and/or substance abuse-related emergency department (ED) visits in 2007 resulted in a hospital admission [5].

Between 1996 and 2006, the number of people with health care expenditures associated with mental disorders nearly doubled from 19.3 million to 36.2 million [6]. While the prevalence of schizophrenia in the U.S. population is only 1%, [7,8] the prevalence is over eight times higher in U.S. Medicaid populations at 8.5% [1]. In Illinois, the prevalence of depression in the general population is about 10.2%; [9] however, the prevalence of depression in the Illinois Medicaid population is more than 39% [1]. Given the high prevalence rates of schizophrenia and depression among Medicaid populations, and the expense in managing these conditions, there is a strong need to help affected individuals better manage their mental health conditions.

### Chronic care model

The chronic care model (CCM) is a framework to guide organizational and quality changes in the health care delivery system to improve the quality of care to people with chronic conditions [10]. The CCM’s focus on the individual’s self-management of chronic conditions was the conceptual prototype for Illinois’ Disease Management (DM) program, called Your Healthcare Plus (YHP)™. This program was offered through the Illinois Department of Healthcare and Family Services and delivered by McKesson Corporation. The CCM paradigm for health care delivery and DM management centers around the partnership between members, staff, and providers, all of whom collaborate for successful disease and self-management education and support [11].

### Disease management

Disease Management emerged as an application of the chronic care model in the mid-1990s, and is used as a strategy to help mitigate costs for health care, particularly the most costly services of inpatient hospital admissions and ED visits [12,13]. While a consensus definition of “disease management” remains ambiguous, [12,14] DM programs do share as their primary objectives the improvement of the quality, consistency and comprehensiveness of cost-effective care for people with chronic illnesses. The shared focus driving all DM programs is improving the health plan member’s ability to self-manage their health condition(s). Thus, DM programs endeavor to shore up member health to avoid costly, unnecessary inpatient hospital admissions, frequent hospital re-admissions, and inappropriate ED utilization. DM program strategies include practices that focus on creating positive sustainable changes in the member's behavior using both educational and motivational strategies to encourage and support goal achievement [15-17].

There is great variation between state Medicaid programs, and DM at the state level is no exception. State Medicaid programs differ in how they select potential DM participants and vary in organizational and structural design. For instance, the diagnosis codes used to identify conditions, the list of conditions to be managed, and the benefit design such as fee-for-service or managed care can vary greatly.

For the Illinois Your Healthcare Plus program, individual health care goals were established in collaboration with participants and their medical homes or primary care physicians. The basic Medicaid benefit package and service access did not change for those participating in the program; medical services were still paid for through Medicaid. Where services such as Assertive Community Treatment were available or where there may have been a practice-based medical home Registered Nurse (RN) care coordinator available, the role of the DM RN was not to take the place of these services, but to support the member’s continued use of these services. It should be noted, however, that due to budgetary constraints for much of the state, often these services were unavailable. Therefore, the focus of the DM RN was to facilitate and support member engagement in any locally available and relevant behavioral and/or medical services.

As many as 38 states have engaged in DM programs for a portion of their Medicaid population, [18] and many self-insured employers have offered DM programs to their employees. Previous dose response literature in the DM domain includes a recent study that found that inpatient admissions dropped by the greatest extent after four or more contacts [19]. This study, however, was limited to people with chronic physical health conditions and not behavioral health conditions, such as schizophrenia and depression.

### Key contribution

The key contribution of this study is to estimate a dose response effect for a DM program designed to improve both care to individuals and cost effective appropriate utilization among Illinois’ Medicaid-only recipients with behavioral health conditions. The literature review found no dose response studies related to a behavioral health DM program. This research is a beginning in filling that knowledge gap.

## Methods

### Ethics statement

Ethics approval/IRB approval was not necessary as the study was performed as part of a DM program using administrative claims data accessible to all authors.

### Subjects

On July 1, 2006, the state of Illinois implemented the Your Healthcare Plus (YHP) DM program, which included the adult aged, blind, and disabled (ABD) Medicaid-only population living in the community and who were non-institutionalized. In fiscal year 2007, the entire Illinois ABD population enrolled in a DM program represented 4.41% of the state’s total Medicaid members, but accounted for over 18% of the state’s Medicaid expenditures, as calculated by Illinois administrative claims analysis.

Subjects were selected based on administrative claims incurred between July 2005 and June 2009 for the ABD Medicaid population living in non-institutional settings. Additionally, subject inclusion criteria required members to have at least two different claims with a diagnosis of depression (DSM-IV criteria and codes for major depressive disorder, dysthymia, and depression not otherwise classified) or schizophrenia. Inclusion criteria for this study also require that subjects were continuously enrolled in Medicaid between July 2005 and June 2009. The total number of subjects with at least one of these conditions or diagnoses and eligible for the intervention was 6,274. Of that number, 1,738 individuals (27.7%) self-selected in to the DM program, were contacted by a nurse, and received interventions at some point between July 2006 and June 2009.

### Intervention

Registered nurses began contacting identified subjects diagnosed with one or both behavioral health conditions (depression or schizophrenia) for DM program enrollment in July 2006. These nurses had at least 5 years of acute care experience and completed 6 weeks of training prior to launch of the DM program. This training included condition-specific information on national clinical guidelines for outpatient management standards, characteristics, and behaviors. Training also included practice in the use of motivational interviewing techniques and concepts [20,21] that help successfully engage members and support them in making better health choices. Furthermore, the training included numerous self-study modules, including communication techniques, cultural sensitivity and awareness, required competencies, and monthly physician-led clinical patient review sessions.

Nurse-led interventions focused on enabling the individual to change their behaviors for improved health and well-being. Nurses provided support regarding lifestyle choices, appropriate use of a medical and/or behavioral therapy home, medication adherence, and participation in other behaviors and habits such as smoking or dietary patterns that may negatively impact well-being.

Members were not randomized into an intervention or control group. For those members who chose to enroll and were able to be contacted, the YHP program customized a self-management intervention plan that included risk stratification, planned education and counseling sessions where nurses worked with participants to help them understand specific disease management skills and behaviors, 24-hour access to nurse counseling, and other sources of condition or symptom advice including telephonic support, printed action plans, and workbooks. In addition, participants received individualized assessment letters and reminders for medication compliance and vaccination. Physicians received alerts about critical signs and symptoms of decompensation and notification of gaps between participant-reported practices and guideline recommendations.

Whether they participate in a DM program or not, health plan members very commonly present with multiple co-occurring physical and behavioral health conditions. The DM program interventions were designed to address both types of conditions by assessing each individual to determine what was most pressing ‘at the moment,’ recognizing the interplay between the conditions and the mind-body relationship.

Participating members received multiple contacts throughout the course of the intervention period. A contact was any interaction related to the participant’s condition. Examples of contacts up to and including the month of measurement are described below and include (1) health assessments (initial, biannual, and annual); (2) monitoring and educational contacts; and (3) inbound symptomatic contacts by participants. Doses were defined as the number of cumulative contacts of any kind that a participant had up to, and including, the month of analysis.

### Member assessments

The nursing assessments (initial, biannual, and annual) included gathering participating members’ self-reported information on areas such as medication use, adherence to their physician’s recommendations, barriers to care, knowledge of their condition(s) and symptoms, current self-management practices, use of a medical home, and recent ED or inpatient utilization. This self-reported information was included in each member’s record. Assessments were always conducted by an RN. While most assessments were done telephonically, some were conducted face-to-face at a mutually agreed upon location with the participating member. An assessment could have been completed in a single session or in more than one session, depending on the willingness of the member. In the case of an incomplete assessment, the nurse would schedule a follow-up session and gather the remaining information. Upon completion of the assessment, an individualized care plan was created.

Assessments occurred initially upon enrollment of a member into the DM program and thereafter at six month intervals to update member status data and to update or revise the care plan and member goals. Providers received a summary letter of member self-reported data along with information on how to contact the DM nurse. Nursing staff also made regular visits to provider practices to collaborate on member management.

### Member monitoring/education contacts

The monitoring and education contacts were scheduled sessions that occurred in the interim period between assessments. Activities typical of this type of contact included addressing the participating member’s care plan challenges and any other pressing member concerns. Nursing staff used motivational interviewing techniques to facilitate needed behavior changes for the participant to be able to effectively self-manage their condition(s).

Condition-specific education was provided and varied depending on the member’s specific needs. Educational content included information to enhance member understanding of their health condition(s), instructions on medication use, guidance on lifestyle choices, and direction on how to recognize symptoms of decompensation and the appropriate actions to take. To support communications between the member and their providers, mock scenarios were play acted between the nurse and members requiring this level of assistance.

While the primary staff delivering interventions in the YHP program were Registered Nurses, other program staff included social workers, community health workers, and behavioral health specialists.

### Unscheduled symptomatic member contacts

Inbound symptomatic member contacts were unscheduled telephonic communications that occurred in addition to the scheduled monitoring and education contacts described above. These inbound contacts occurred when a participating member reported health symptoms, flagging the nurse to follow up the next day to ensure that the appropriate steps had been taken. This contact type was also used to address other member-reported issues that that were appropriate for follow-up prior to the next scheduled contact. Examples include follow-up with the member after a visit to a provider, and DM staff counseling when a participating member wanted to discuss current symptoms or other urgent needs.

### Research design

Panel/longitudinal data was used for this analysis. The unit of measurement was the individual Medicaid member month with each participating member measured for each calendar month between July 2005 and June 2009. All members in the analysis were continuously eligible for DM services between July 2005 and June 2009. Because the intervention began in July 2006, the 12 months of data between July 2005 and June 2006 did not have any DM program contacts for any members.

Given the panel data structure of one observation for each member for each month of their Medicaid eligibility, a member could have switched from a non-intervention status to an intervention status for subsequent months. That is, since each member was continuously eligible for the entire 48 month period, if the member began receiving intervention contacts in month 37, then only months 37–48 were logged as months with an indicated contact. Once a member started receiving intervention contacts, every subsequent month was indicated as a month of contact, since the contacts in each month are the cumulative number of contacts a member has had up to that point.

Multivariate regression analysis of panel data was used to evaluate the intervention and test the hypothesis that increased DM contacts lowered the odds of an inpatient admission or emergency room visit. The dependent variable was the dichotomous indicator as to whether or not the member had an inpatient admission in a given month, or had an ED visit for those separate regressions. For each monthly observation, an indicator of whether or not an inpatient admission or ED visit occurred during that month was calculated. The dichotomous formulation of this variable allows for a logistic regression to be estimated and odds ratios to be calculated.

The independent or explanatory variables included (i) an identification indicator showing whether or not a member had been identified for a particular DM intervention or plan of care for their depression or schizophrenia, (ii) the cumulative number of contacts (doses) for each particular intervention a member has had, including doses squared and cubed, (iii) a high risk indicator, defined as members identified as having cancer, end stage renal disease, HIV, hemophilia, traumatic brain injury or a previous organ transplant, (iv) age/gender grouping, (v) the predictive model risk score prior to identification of each member for a DM program calculated by the predictive modeling company MEDai, [22] and (vi) a time variable which was incremented by one for each month to account for potential trends.

Multivariate regression analysis allowed for simultaneous estimation of several explanatory variables influencing a participating member either at the same or different times. That is, if a member had both depression and schizophrenia, the number of contacts related to each condition was used as an explanatory variable. In cases such as this, there was one explanatory variable for the cumulative number of depression contacts up to that month, as well as one explanatory variable for the cumulative number of schizophrenia contacts up to that month.

To calculate the dose response effect, a cubic representation was chosen to allow for a flexible functional representation. This flexibility allowed for a quadratic and linear dose response relationship as a special case. Contact or dose variables were calculated for each condition. For instance, the number of depression contacts, the number of depression contacts squared, and the number of depression contacts cubed was calculated. The coefficients of each of these three variables were then estimated in the regression analysis and used to estimate the odds ratio for each level of contact by exponentiation of the coefficient estimates.

## Results

### Descriptive statistics

Table 1 shows that of the 6,274 members who were diagnosed with either depression or schizophrenia, 1,738 (27.7%) were managed through direct nurse contact for at least one month at some point over the 3 year intervention time period. All participating members were continuously enrolled for the entire 48 months, which included the 12 months prior to the start of the intervention. High risk members accounted for 5.6% of the population, while 3.9% of the members were identified for both the depression and schizophrenia programs. The annualized inpatient admission rate per 1,000 members was 671.1, and the annualized ED utilization rate was 1,793.1, indicating that these members were heavy users of the health care system. In addition, face-to-face contacts accounted for less than 1% for all contact types for both conditions, except for schizophrenia symptomatic contacts which accounted for 3.6% of contacts.

**Table 1:** Descriptive statistics

	**Total eligible population**	**Participants**	**Non-participants**
Unique Members	6,274	1,738	4,536
Members receiving intervention (%)	27.7	100.0	0.0
Members eligible all 48 months (%)	100	100	100
High Risk Members (%)	5.6	6.2	5.4
Risk Score (Mean)	33.1	32.9	33.1
Female (%)	60.2	60.2	60.2
Female age less than 29 (%)	7.4	6.0	7.9
Female age 30–39 (%)	11.5	10.0	12.1
Female age 40–49 (%)	21.0	21.5	20.9
Female age 50+ (%)	20.3	22.7	19.4
Male age less than 29 (%)	9.0	8.2	9.2
Male age 30–39 (%)	8.5	8.5	8.5
Male age 40–49 (%)	14.0	14.1	14.0
Male age 50+ (%)	8.4	9.0	8.2
Annualized Inpatient Admissions per 1,000 members	671.1	396.6	776.2
Annualized ER Visits per 1,000 members	1,793.1	1,514.1	1,900.0
People identified for both programs (%)	3.9	10.4	1.5

### Multivariate regression results

**Figure 1:**
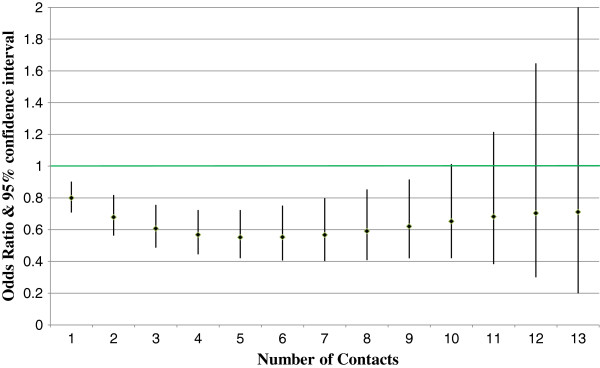
**Dose response impact for depression inpatient admissions.** Diamonds represent the odds ratio estimate. The lines represent the 95% confidence interval. Those number of contacts with a confidence interval that does not cross the green line are statistically significant.

**Figure 2:**
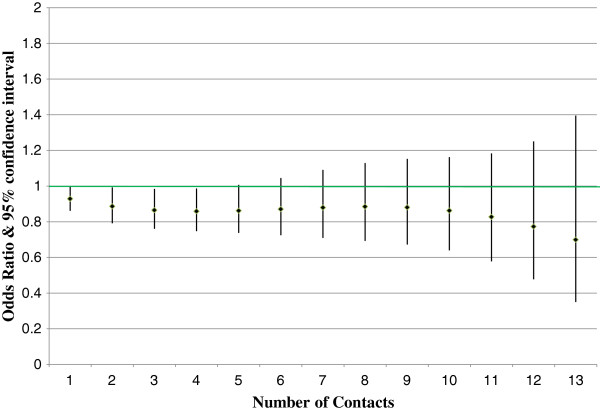
**Dose response impact for depression emergency department visits.** Diamonds represent the odds ratio estimate. The lines represent the 95% confidence interval. Those number of contacts with a confidence interval that does not cross the green line are statistically significant.

**Figure 3:**
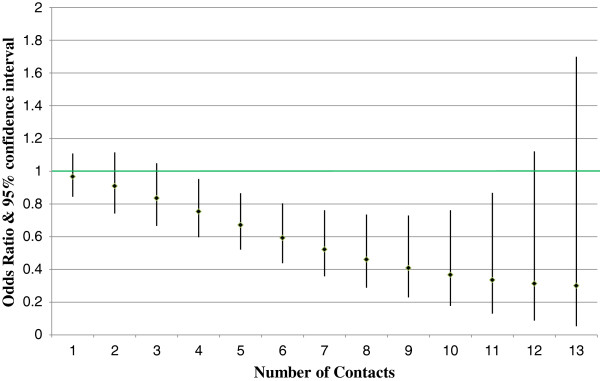
**Dose response impact for schizophrenia inpatient admissions.** Diamonds represent the odds ratio estimate. The lines represent the 95% confidence interval. Those number of contacts with a confidence interval that does not cross the green line are statistically significant.

**Figure 4:**
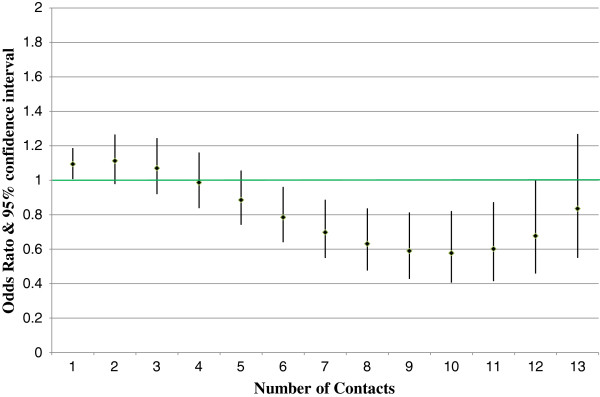
**Dose response impact for schizophrenia emergency department visits.** Diamonds represent the odds ratio estimate. The lines represent the 95% confidence interval. Those number of contacts with a confidence interval that does not cross the green line are statistically significant.

The dose response impact is considered statistically significant (rejecting the null hypothesis that the dose variable odds ratio is 1.0) if Pearson’s chi-square test p-value is less than the critical value. Figures 1 and 2 show the all-cause inpatient admission and ED visit results by dose (nurse contact) level for members with depression. Figures 3 and 4 show similar all-cause inpatient admission and ED visit results for members with schizophrenia. The points in each figure represent the odds ratio, whereas the lines indicate the 95% confidence interval. If the confidence interval contains the number 1, then that particular odds ratio is not statistically significant at the 5% level. Table 2 shows the same odds ratios and levels of statistical significance for each level of contact for critical values of 1%, 5%, and 10%.

**Table 2:** Odds ratios by condition by number of doses

	**Inpatient admissions**		**ED visits**	
**Doses**	**Depression**	**Schizophrenia**	**Depression**	**Schizophrenia**
1	0.80***	0.97	0.93*	1.09**
2	0.68***	0.91	0.89**	1.11
3	0.61***	0.84	0.86**	1.07
4	0.57***	0.75**	0.86**	0.99
5	0.55***	0.67***	0.86*	0.89
6	0.55***	0.59***	0.87	0.78**
7	0.57***	0.52***	0.88	0.7***
8	0.59***	0.46***	0.88	0.63***
9	0.62**	0.41***	0.88	0.59***
10	0.65*	0.37***	0.86	0.58***
11	0.68	0.34**	0.83	0.6***
12	0.70	0.31*	0.77	0.68**
13	0.71	0.30	0.70	0.83

*P-value < = 0.10.

**P-value < = 0.05.

***P-value < = 0.01.

For participating members with depression, all-cause inpatient admissions showed statistically significant impact from one to nine contacts with odds ratios between 0.8 and 0.62. Depression DM program impact on emergency room utilization was lower than the program impact on inpatient admissions. Results were statistically significant from two to four contacts, with odds ratios ranging from 0.89 to 0.86.

For schizophrenia, all-cause inpatient admissions showed statistically significant impact from four to eleven contacts with odds ratios between 0.75 and 0.34. Similar to the above finding for the depression DM program, schizophrenia DM program impact on emergency room utilization was also lower than the program impact on inpatient admissions. Results were statistically significant from six to twelve contacts, with odds ratios ranging from 0.78 to 0.68.

For both depression and schizophrenia, the increased number of contacts showed greater impact on reductions in inpatient admissions than it did on the number of ED visits. This indicates that the intervention had less influence in reducing ED visits as compared to inpatient admissions. For participating members with schizophrenia, more contacts were required to show a statistically significant reduction in ED and inpatient admissions as compared to participating members with depression, where impacts were seen with fewer contacts.

## Discussion

Figures 1, 2, 3 and 4 reveal some insights in the management of Medicaid members with depression or schizophrenia. First, the reductions in all-cause inpatient admissions happened sooner for participating members with depression than for participating members with schizophrenia. As such, measurable impacts can be seen faster for those members with depression. Second, for both disease states, higher numbers of contacts were not statistically significant in reducing inpatient admissions or ED utilization. It is doubtful that this is due to a lack of program impact, but rather due to fewer people having very high numbers of contacts. All participants had at least one contact by a DM nurse, but only 2.5% of depression and 0.7% of schizophrenia members had 13 or more contacts. Third, similar to inpatient admissions, impacts in the number of ED visits required a higher number of contacts to show statistically significant reductions. This data reveals that participating members with schizophrenia took longer to show programmatic impacts both in terms of the number of inpatient admissions and in terms of ED visits. This is due to the larger variance in ED utilization measured for participating members with depression. However, although members with depression had statistically significant reductions in the number of ED visits sooner than those members with schizophrenia, the effects diminished faster in terms of statistical significance, due again to the larger variance in the number of ED visits.

One limitation of this study may be its generalizability. This study was specific to a state Medicaid population, and translating these findings to either another Medicaid population or to a commercially insured population may not be valid. This, however, suggests an area for future research. Another limitation is the quasi-experimental study design. Selection bias may exist because members may choose whether to participate in the program for reasons that may not be controlled for with the observable variables. For example, certain members may be more motivated for self-care or have different complexities related to their disease(s). Predictive risk adjustment may be a possible control. As well, even though members agree to participate in the DM program, the level and number of contacts are determined through risk assessment. Apart from an experimental design, the magnitude of this potential bias cannot be determined. A further limitation is restricting the sample to continuously enrolled members. Medicaid members do churn in and out of eligibility; however, continuously enrolled members were chosen to ensure that each person was equally represented and had complete data. Finally, dose timing was not examined here but might provide further insights into the effect of the intervention. Future analyses could examine the differential effects of 12 monthly doses versus 12 doses received over the course of 2 years.

## Conclusions

Program staff cited that the most difficult challenges in delivering effective DM programs stem from the participating populations’ burden of illness, dysfunctional patterns of health behaviors, and years of barriers to access to care that have contributed to unfortunate decision making in their own health care and lifestyle choices. Due to both the varied nature of human behavior and the complexity of chronic health problems, achieving a positive impact on health outcomes and behaviors must rely on a multipronged approach that focuses on supporting and motivating the individual member. For members with chronic mental illnesses, a caring, individualized approach provides the attention that they may not otherwise perceive as being provided.

This study has evaluated a multipronged approach focusing on supporting and motivating the individual member. A thorough analysis of the program data found statistically significant reductions of all-cause inpatient hospitalizations and ED visits for Medicaid members diagnosed with depression or schizophrenia. These reductions were based on the number of contacts and were not the result of denying inpatient utilization or restricting treatment.

This analytical work has been valuable for Illinois Medicaid and the Your Healthcare Plus program by providing data-driven information that may be used in making program modifications and staffing adjustments. Program changes suggested by these results include altering the schedule of contacts and ensuring both regular and ad hoc contacts to meet participating member needs. It is important that resources be allocated for interventions where the best opportunities for improvements in DM program outcomes may exist. As this is the first dose response study related to a non-drug DM intervention for behavioral health in the literature, additional future studies are needed in this area to determine further methods of improving health outcomes.

## Competing interests

WM and MM have no conflict of interest. GDB, SD, and KW work for McKesson Corporation, which provided services to the state of Illinois.

## Authors’ contributions

GDB and SD have made substantial contributions to conception and design. GDB and SD performed the data analysis. GDB, SD, KW, WM and MM were involved with interpretation of data. GDB, SD, KW, WM and MM have been involved in drafting the manuscript or revising it critically for important intellectual content. GDB, SD, KW, WM and MM have all given final approval of the version to be published. All authors read and approved the final manuscript.

## Pre-publication history

The pre-publication history for this paper can be accessed here:

http://www.biomedcentral.com/1472-6963/14/288/prepub
